# Molecular Prevalence of *Anaplasma marginale* and *Ehrlichia* in Domestic Large Ruminants and *Rhipicephalus* (*Boophilus*) *microplus* Ticks From Southern Luzon, Philippines

**DOI:** 10.3389/fvets.2021.746705

**Published:** 2021-10-13

**Authors:** Remil L. Galay, Carina R. Llaneta, Maria Karla Faye B. Monreal, Antero L. Armero, Arianne Bel D. Baluyut, Czarina Marie F. Regino, Kristina Andrea C. Sandalo, Billy P. Divina, Melbourne R. Talactac, Lennox P. Tapawan, Maarten Czar L. Mojares, Cherry R. Alvarez, Emmanuel R. Mago, Noemi D. Encarnacion, Masako Andoh, Tetsuya Tanaka

**Affiliations:** ^1^Department of Veterinary Paraclinical Sciences, College of Veterinary Medicine, University of the Philippines Los Baños, College, Los Baños, Philippines; ^2^Department of Agriculture Regional Field Office 3, San Fernando, Philippines; ^3^Department of Clinical and Population Health, College of Veterinary Medicine and Biomedical Sciences, Cavite State University, Indang, Philippines; ^4^Laboratory of Public Health, Joint Faculty of Veterinary Medicine, Kagoshima University, Kagoshima, Japan; ^5^Laboratory of Infectious Diseases, Joint Faculty of Veterinary Medicine, Kagoshima University, Kagoshima, Japan

**Keywords:** *Anaplasma marginale*, *Ehrlichia*, cattle, *Rhipicephalus (Boophilus) microplus*, tick- borne rickettsiae, water buffalo

## Abstract

Anaplasmosis and ehrlichiosis are tick-borne rickettsial diseases that cause significant economic losses in the livestock industry worldwide. Although bovine anaplasmosis is known to be endemic in the Philippines, epidemiological data is fragmented. Moreover, little is known about bovine ehrlichiosis in the country. In this study, the prevalence of *Anaplasma marginale* and *Ehrlichia* in cattle and water buffalo from provinces in the southern part of Luzon, Philippines, was investigated through PCR. Blood samples from 620 animals comprised of 512 cattle and 108 water buffalo and 195 tick samples were subjected to nested PCR targeting the *groESL* gene of Anaplasmataceae. Positive samples were further subjected to another nested PCR and conventional PCR to amplify the *A. marginale groEL* gene and the *Ehrlichia dsbA* gene, respectively. Selected *A. marginale*-positive samples were also subjected to nested PCR targeting the *msp5* gene. Regardless of the animal host, the overall prevalence in blood samples obtained was 51.9% for Anaplasmataceae, 43% for *A. marginale*, and 1.1% for *Ehrlichia*. No water buffalo were positive for *Ehrlichia*. Meanwhile, 15.9, 6.7, and 2% of the tick samples, all morphologically identified as *Rhipicephalus* (*Boophilus*) *microplus*, were positive for Anaplasmataceae, *A. marginale*, and *Ehrlichia*, respectively. Sequence analysis of selected *A. marginale msp5* amplicons showed that the isolates from the region share 94–98% identity to reported *A. marginale* from other countries. The phylogenetic tree showed clustering of isolates in the region and a close relationship with *A. marginale* isolates from other countries. Sequences of *Ehrlichia* amplicons from cattle and ticks were 97–100% similar to reported *Ehrlichia minasensis* isolates. This study showed the high prevalence of *A. marginale* in Luzon, Philippines, and provided the first molecular evidence of *E. minasensis* in the country.

## Introduction

Tick-borne diseases (TBDs) cause significant economic losses to the livestock industry worldwide, particularly in tropical and subtropical regions (1). The global annual losses in the cattle industry attributed to ticks and TBDs have been estimated to be between US$ 14 to US$ 19 billion (2). Anaplasmosis and ehrlichiosis are rickettsial TBDs caused by members of the family Anaplasmataceae. Bovine anaplasmosis due to *Anaplasma marginale* is considered the most prevalent TBD of cattle. Hard ticks under the genus *Rhipicephalus* (*Boophilus*) primarily transmit *A. marginale* (3), but it can also be transmitted by blood-sucking flies and sucking lice, as well as contaminated fomites (4). Meanwhile, bovine ehrlichiosis is caused by several species of *Ehrlichia*, also mainly transmitted by several hard ticks. The common species affecting large ruminants are *Ehrlichia bovis, E. ondiri, E. chaffeensis*, and *E. ruminantium* (5). These pathogens are distributed worldwide but more widely distributed in European countries, India, and South Africa. Additionally, a new species of *Ehrlichia* was isolated from the hemolymph of *R. microplus* and was named *E. mineirensis* (6). Using PCR, another genotype, UFMT-BV, was later detected in naturally infected cattle from Brazil. Through the genetic characterization of 16S ribosomal RNA (*16s rRNA*) and thio-disulfide oxidoreductase (*dsb*) genes, it was later found that the two genotypes represented a single species phylogenetically close to *E. canis* that was named *E. minasensis* (7).

The cattle industry is a developing agricultural sector in the Philippines. In 2020, a population of 2.63 million with a gross value of about US$582 million was reported in the country (8). Water buffalo, which are raised for draft power, meat, and milk, had a reported population of 2.83 million with a gross value of about US$260 million in 2020 (9). Despite continued efforts to boost the large ruminant industry, particularly the production of dairy cattle and water buffalo, health problems, including tick infestation, still hamper high productivity. The tropical climate of the Philippines highly favors the life cycle of the cattle tick *R. microplus*. The wide distribution of this tick in the country is accompanied by the occurrence of TBDs, such as anaplasmosis (10). Several studies provided molecular evidence of the occurrence of *A. marginale* in cattle (11–13) and water buffalo (14, 15) in the Philippines. High genetic diversity of *A. marginale* was also reported based on the analysis of the *msp1a* gene (16). Nevertheless, epidemiological data in the country remains fragmented. Aside from its presence in the blood, we previously reported the detection of *A. marginale* in the milk of dairy cattle (17). A study previously reported the detection of *Ehrlichia* in 35 of 246 bovine blood samples through PCR, but the species was not identified (18). Here, we investigated the epidemiology of *A. marginale* and *Ehrlichia* in cattle, water buffalo, and *R. microplus* ticks from CALABARZON (Region IV-A) in Luzon, Philippines—a top cattle-producing region, also with a large population of water buffalo.

## Materials and Methods

### Blood and Tick Samples

A total of 620 blood samples (512 cattle and 108 water buffalo) and 195 tick samples comprised of various developmental stages were tested in this study by PCR for the presence of Anaplasmataceae bacteria, particularly *A. marginale* and *Ehrlichia*. The number of blood samples sufficient to determine the prevalence in animals was calculated based on the animal population data in the study area at 95% confidence interval using an online software (https://www.openepi.com). The samples were collected between March 2016 and October 2019 from selected commercial farms and smallholder raisers in 44 municipalities/cities throughout the five provinces (Cavite, Laguna, Batangas, Rizal, and Quezon) of the CALABARZON region (Region 4A) in Luzon, Philippines as previously described (19). The animals were of various breed type, age and sex, and most of which are not showing any signs of severe disease at the time of sample collection. All ticks were collected directly from the animals and were morphologically identified as *R. microplus*. DNA was extracted from blood and tick samples using commercial extraction kits (innuPREP^®^ DNA/RNA Mini Kit for blood and blackPREP^®^ Tick DNA/RNA Kit, Analytik Jena, Jena, Germany).

### PCR Detection of *Anaplasma* and *Ehrlichia*

Sample screening was initially done through nested PCR amplification of the *groESL* gene of Anaplasmataceae using the primers described by Tabara et al. (20). Samples that showed positive bands were subjected to nested PCR targeting the heat-shock operon (*groEL*) gene for *A. marginale* (12) and conventional PCR targeting the *dsb* gene of *Ehrlichia* ((34)). Selected samples positive for *A. marginale* were also subjected to nested PCR targeting the *msp5* gene ((35)). All primers used in this study are listed in Supplementary Table 1. PCR mixtures with a total volume of 10 μl were prepared using Tks Gflex™ DNA Polymerase (TaKaRa, Shiga, Japan), together with 10 pmol each of forward and reverse primers, nuclease-free water, and template (1 μl DNA or 1^st^ PCR product). The PCR conditions are shown in Supplementary Table 2. In each PCR batch, a negative control containing nuclease-free water was included. PCR products were subjected to electrophoresis in 2% agarose gel in 1x TAE buffer. After staining the gel with ethidium bromide in 1x TAE, the bands were visualized through a gel documentation system (Bio-Print, Vilber, Lourmat, France).

### Sequence and Data Analysis

Selected *A. marginale msp5*- and *Ehrlichia dsbA-*positive samples were subjected to PCR at 50 μl mixtures for sequence reading. The amplicons were purified using NucleoSpin^®^ Gel and PCR Clean-up kit (Macherey-Nagel, Leicestershire, England) following the manufacturer's protocol. Capillary sequencing was accomplished by a third-party laboratory using the forward primer for nested PCR. The sequences for each gene were aligned using Clustal Omega software (https://www.ebi.ac.uk), and the percent identity was determined. Sequence readings were compared to sequences of reported isolates using the Basic Local Alignment Search Tool or BLAST^®^ (https://blast.ncbi.nlm.nih.gov/Blast.cgi). A maximum likelihood phylogenetic tree was constructed using MEGA v.7 software, with bootstrap values estimated using 1,000 replicates based on Kimura's two-parameter substitution model (K2P distance). The prevalence of each pathogen in animals was calculated by dividing the number of positive samples by the total number of blood samples and per animal, expressed as a percentage. The positivity rate in ticks was also calculated and expressed as percentage. The occurrence of *A. marginale* based on host attributes including species, purpose, and sex was also calculated, and chi-square analysis at a 95% confidence interval (α = 0.05) was performed using the online software WinEpi^®^ to determine the presence of association.

## Results

### PCR Detection

Table 1 shows the results of PCR detection of *A. marginale* and *Ehrlichia* in blood samples from cattle and water buffalo. Initial screening for Anaplasmataceae through nested PCR targeting the *groESL* gene was performed, which revealed 322 (51.9%) positive samples, of which 309 (60.3%) were from cattle and 13 (12%) were from water buffalo. These positive samples were then subjected to nested PCR for the *A. marginale groEL* gene, which revealed an overall prevalence of 43%. According to the host, 266 cattle were positive, with a prevalence of 51.9%, while only one water buffalo (0.9%) was *A. marginale* positive. Regarding the type of animal, a higher prevalence of 63.8% was observed among dairy animals than beef-type animals that mainly were native Philippine cattle. Meanwhile, the prevalence of *A. marginale* among males and females was almost equal. Chi-square analysis showed significant association (*p* < 0.0001) of *A. marginale* infection with host species and type (Table 2).

**Table 1:** Prevalence of *A. marginale* and *Ehrlichia* in cattle and water buffalo from the CALABARZON region in Luzon, Philippines, based on PCR detection in blood samples.

**Province**	**Cattle**				**Water buffaloes**			
	***N***	**No. (%) positive for Anaplasma-taceae**	**No. (%) positive for *A. marginale***	**No. (%) positive for *Ehrlichia***	***n***	**No. (%) positive for Anaplasma-taceae**	**No. (%) positive for *A. marginale***	**No. (%) positive for *Ehrlichia***
Cavite	100	73 (73)	66 (66)	1 (1)	0	–	–	–
Laguna	111	68 (61.3)	61 (54.9)	1(0.9)	11	1 (9)	0	0
Batangas	120	85(70.8)	71 (59.2.)	0	8	7 (87.5)	1 (12.5)	0
Rizal	87	32 (35.63)	27 (31.03)	0	0	–	–	–
Quezon	94	51 (54.3)	41 (43.6)	5 (5.32)	89	5 (5.6)	0	0
Total	512	309 (60.3)	266 (51.9)	7 (1.4)	108	13 (12)	1 (0.9)	0

**Table 2:** Occurrence of *A. marginale* with regard to host attributes.

**Host**	***n***	**Number (%) positive**	***p*-value**
**attribute**		**for *A. marginale***	
**Species**			
Cattle	512	266 (51.9)	<0.0001*
Water buffalo	108	1 (12.5)	
**Type**			
Dairy	268	171 (63.8)	
Beef	284	94 (33.1)	<0.0001*
Draft	68	1 (1.5)	
**Sex**			
Male	110	42 (38.2)	
Female	510	207 (40.6)	0.6405

*Chi-square analysis was done to determine the presence of association, and p-values are shown. Asterisk indicates statistical significance (p < 0.05)*.

Meanwhile, only seven blood samples, all from cattle, were found positive through conventional PCR for the *Ehrlichia dsbA* gene, resulting in an overall prevalence of 1.1%. PCR detection in tick samples showed that 31 of 195 (15.9%) were positive for Anaplasmataceae (Table 3). Further PCR analyses revealed that 13 (6.7%) and 4 (2%) samples were positive for *A. marginale* and *Ehrlichia*, respectively. Almost all of the positive samples were female ticks.

**Table 3:** Positivity rate (%) of *A. marginale* and *Ehrlichia* in ticks from the CALABARZON region in Luzon, Philippines.

**Province**	***N***	**No. (%) positive for Anaplasmataceae**	**No. (%) positive for *A. marginale***	**No. (%) positive for *Ehrlichia***
Cavite	89	0	–	–
Laguna	18	3 (16.7)	2 (11.1)	0
Batangas	50	18(36)	6 (0.12)	0
Rizal	0	–	–	–
Quezon	38	10 (26.3)	5 (13.2)	4 (10.5)
Total	195	31 (15.9)	13 (6.7)	4 (2)

### Sequence Analysis

**Figure 1:**
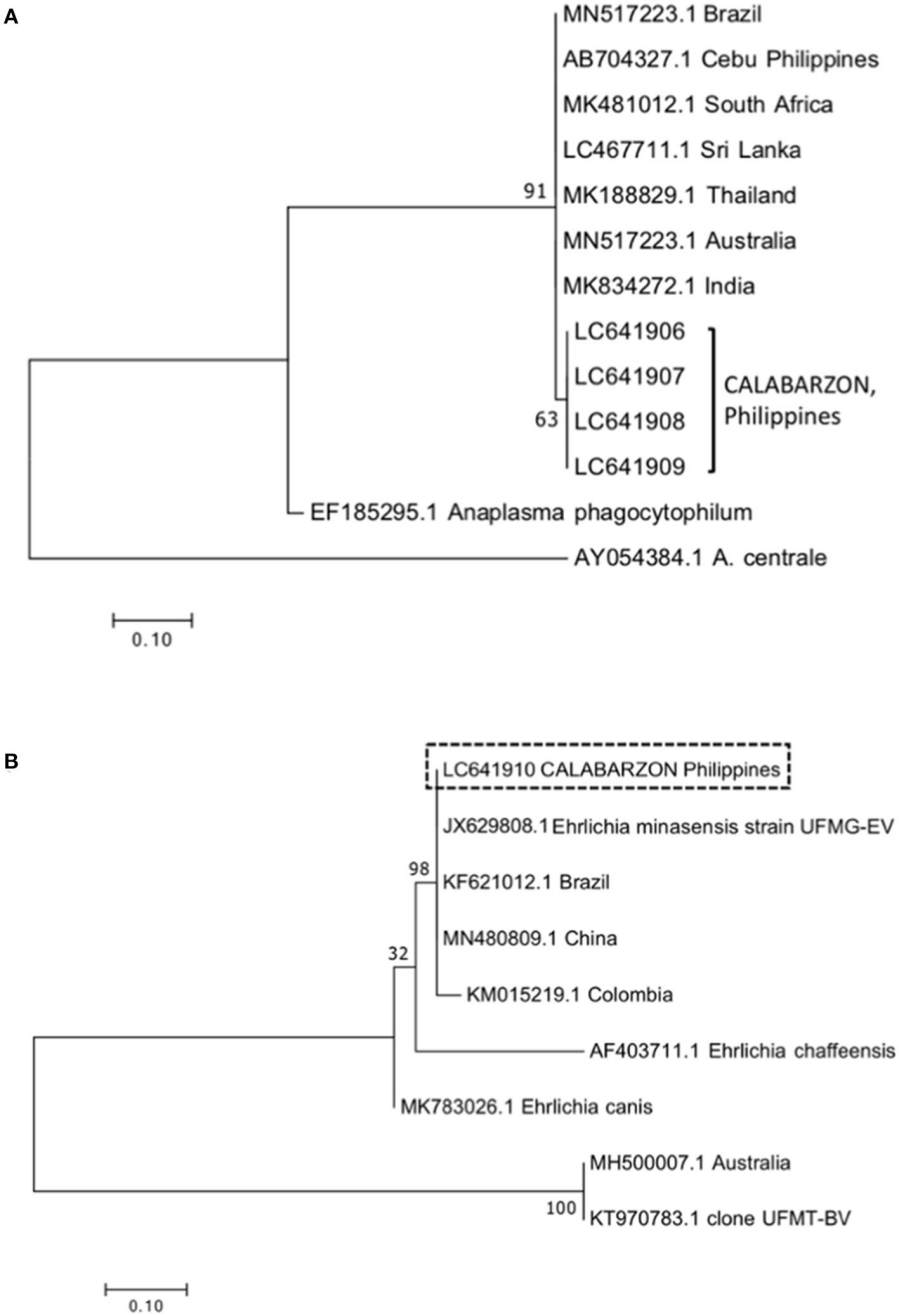
Phylogenetic trees for *Anaplasma marginale msp5* **(A)** and *Ehrlichia dsbA* **(B)** constructed using the maximum likelihood method. Numbers in the nodes indicate support values based on Kimura's two-parameter substitution model (K2P distance). Isolates from this study are indicated within brackets **(A)** or enclosed within a box **(B)**. The bar represents 0.10 substitutions per site.

Ten *A. marginale msp5* amplicons from blood samples (two from each province) were subjected to sequence analysis. After multiple sequence alignment of the 160-bp fragment, it was found that the amplicons per province were 98–100% similar, and the amplicons from all provinces shared 97–100% identity. The reported isolate from another island of the Philippines, Cebu, had a homology with the amplicons ranging from 95–98%. BLAST analysis showed the amplicons share 96–99% identity with reported *A. marginale* isolates from Australia (MN517223.1), India (MK834272.1), Thailand (MK188829.1), Sri Lanka (LC467711.1), South Africa (MK481012.1), and Brazil (MN517223.1). The phylogenetic tree showed the clustering of amplicons from this study in a single branch (Figure 1A). The tree also showed a close relationship with but branching separate from other reported isolates, including that from Cebu, Philippines. The sequences of four *A. marginale msp5* amplicons were deposited in the DNA Data Bank of Japan (accession numbers LC641906–LC641909).

Meanwhile, four *Ehrlichia* amplicons (two blood samples and two tick samples) were sequenced. The alignment of multiple nucleotide sequences of the 350-bp fragment showed that the identity was 99–100%. Based on BLAST analysis, the amplicons were 99% identical to *Ehrlichia* isolated from *R. microplus* ticks, designated as *E. minasensis* strain UFMG-EV (JX629808.1), and share 97–100% identity with reported *E. minasensis* isolates from Brazil (KF621012.1), Colombia (KM015219.1), and China (MN480809.1). A single sequence was deposited in the DNA Data Bank of Japan (accession number LC641910). The phylogenetic tree showed the clustering of amplicons with the abovementioned *E. minasensis* isolates in a single branch (Figure 1B).

## Discussion

Anaplasmosis and ehrlichiosis are tick-borne rickettsial diseases that can have a detrimental impact on the health and productivity of livestock worldwide. Previous studies in the Philippines showed the widespread distribution of *A. marginale* in cattle and water buffalo, yet epidemiological data is still fragmented. Meanwhile, the occurrence of *Ehrlichia* in cattle, water buffalo, and *R. microplus* ticks in the country has not been established. Thus, this study was conducted to determine the occurrence of *A. marginale* and *Ehrlichia* in cattle, water buffalo, and *R. microplus* ticks in the CALABARZON region, which ranks third and ninth in terms of cattle and water buffalo populations, respectively (PSA 2020).

A high prevalence of *A. marginale* was observed in cattle, but it was lower than the prevalence previously reported in a study in Luzon involving two dairy farms (13) and the detection rate in our study in the same region that only included milking dairy cattle (17). The current study included dairy cattle, most of which are Holstein Friesian or Holstein-Sahiwal crosses, as well as the tropical native Philippine cattle commonly raised for beef. The latter breed is known to be tick-resistant. However, our results show that the native cattle can still harbor the pathogen and become an important source of infection under field conditions. A similar prevalence of *A. marginale* in beef cattle was also reported in Thailand (21). The high occurrence in dairy cattle agrees with the results of the above-mentioned studies. It was noted during field collection that *R. microplus* ticks are more commonly encountered in dairy cattle than in beef cattle. Interestingly, no significant clinical signs were observed from infected cattle, except for a few that were emaciated. It is also possible that most of these *A. marginale*-positive animals are already in the carrier state (22). These carrier animals can, however, pose a problem when introducing naïve animals to the farm, with either breed being at risk of severe disease if exposed to virulent *A. marginale* (23).

Water buffalo, which are mostly utilized as draft animals, had a remarkably lower prevalence of *A. marginale* infection than cattle. Although there was a large difference in the number of samples in this study, this finding may also be explained by the innate resistance of water buffalo to tick infestation due to their thicker skin and habit of submerging in wetlands, which prevents tick attachment (24). While the cattle tick *R. microplus* is also listed as one of the ectoparasites of water buffalo (32), this was rarely observed in water buffalo during our field collection. An experimental study confirmed the potential of water buffalo as hosts of *R. microplus* ticks (25). Thus, water buffalo may act as important reservoirs for the cattle tick and the pathogens that it transmits. Moreover, other blood-sucking arthropods, such as the louse *Haematopinus tuberculatus* which was commonly observed among the water buffalo during sample collection, can transmit *A. marginale* (24, 26). Meanwhile, the obtained prevalence in this study was notably lower than that of previous reports in water buffalo from Luzon (14) and Bohol, another island in the Philippines (15).

Moreover, a significantly higher number of females were infected with *A. marginale* as compared to males. This might be due to their greater susceptibility to infection caused by hormonal disturbances during pregnancy, parturition, and lactation, which causes stress and immunosuppression, especially in high-producing cows. Furthermore, imbalances in progesterone, estrogen, and cortisol serum levels contribute to the impairment of the immune function of females (27). It is important to note that most of the animals included in the study were lactating dairy animals. The apparent carrier state of dairy females, suggested by the absence of apparent clinical signs at the time of blood collection, still poses a risk for clinical disease since relapse is possible following immunosuppression (28).

On the other hand, *Ehrlichia* was detected in some blood samples and ticks from cattle. Most of the cattle found positive for *Ehrlichia* were female dairy cattle, none showing any clinical signs. Interestingly, none of the positive animals were observed to have ticks at the time of blood collection, suggesting possible chronic or carrier status. Based on sequence analysis, the species detected was *Ehrlichia minasensis*. Similar to the detection rate obtained in this study, ((33)) also reported a low detection rate of *E. minasensis* (previously designated as UFMT-BV) among Brazilian cattle, mostly dairy cattle. Additionally, a low detection rate was also reported among cattle in Ethiopia (29). Phylogenetic analysis showed that the isolate found in this study was closely related to reported isolates of *E. minasensis* from other countries, suggesting the widespread occurrence of this rickettsial organism.

A great majority of the animals included in this study did not have ticks during blood collection, hence, the discrepancy in the number of blood and tick samples. Most commercial farms included in this study are using chemical acaricides to control tick infestation. The positivity rate in ticks was also lower than in blood, similar to a previous report in Malaysia (30). There were ticks collected from animals whose blood tested positive for *A. marginale* or *Ehrlichia*, suggesting that the tick did not yet acquire the pathogen or the bacterial load in the ticks was below the detection limit of the PCR assays.

Analysis of the *msp5* gene confirmed the close relationship of *A. marginale* isolates in this study with isolates from another island of the Philippines, Cebu, and other countries. A high diversity of *A. marginale* was observed in a previous study based on the analysis of the *msp1a* gene of samples from several provinces in the Philippines, including Cavite and Batangas, which are covered in this study (16). In contrast, the phylogenetic tree based on the *msp5* gene showed the clustering of isolates from different provinces in this study in a single branch, indicating that the gene is conserved among those isolates. On another note, some animals positive for the *groESL* gene of Anaplasmataceae did not turn positive for either *A. marginale* or *Ehrlichia*, suggesting possible infection with another species, such as *A. centrale*. Furthermore, some commercial farms reported having dairy cattle vaccinated against anaplasmosis, which utilized live *A. centrale* (31). Although we were unable to confirm it from the farm records, there is a possibility that those animals positive for *Anaplasma groESL* but negative for *A. marginale* and *Ehrlichia* were vaccinated with live *A. centrale*. Future studies should investigate the occurrence of other *Anaplasma* species through PCR assays that are species-specific.

To conclude, this study showed the high prevalence of *A. marginale* in cattle in southern Luzon, Philippines, and confirmed the presence of *E. minasensis* in naturally-infected cattle and *R. microplus* ticks in the country. Although a more significant number of dairy cattle were found to be infected with either pathogen, the detection of the pathogens in native beef cattle implies the latter's role in maintenance and transmission in the field. The result of our study adds knowledge regarding the epidemiology of *A. marginale* and the geographical location of *E. minasensis*, which is a potential emerging pathogen of cattle. Further studies on the epidemiology of *E. minasensis* in the Philippines are necessary. The effects of *E. minasensis* in the health and productivity of cattle should also be investigated further since clinical manifestations were observed after experimental infection in a calf ((33)). The findings of this study highlight the need for continued surveillance and intensified control programs against rickettsial TBDs in the Philippines.

## Data Availability Statement

The original contributions generated for the study are included in the article/Supplementary Files, further inquiries can be directed to the corresponding author/s.

## Ethics Statement

The animal study was reviewed and approved by Institutional Animal Care and Use Committee (IACUC) of the College of Veterinary Medicine, University of the Philippines Los Baños. Written informed consent was obtained from the owners for the participation of their animals in this study.

## Author Contributions

RG, BD, MT, and TT conceptualized the study. RG, CL, MarM, AA, AB, CR, KS, LT, and MaaM collected the samples and conducted the laboratory experiments. RG, BD, MT, CA, EM, and NE supervised the work. RG wrote the original draft of the manuscript. RG and TT acquired funding for the study. All authors analyzed the data, reviewed and approved the final version of the manuscript.

## Funding

This research was funded by the University of the Philippines Balik Ph.D. (Foreign-trained Ph.D.) grant, the Japan Society for the Promotion of Science grant numbers 15H05264 and 20KK0154, and JSPS Bilateral Program grant number JPJSBP120219936.

## Conflict of Interest

The authors declare that the research was conducted in the absence of any commercial or financial relationships that could be construed as a potential conflict of interest.

## Publisher's Note

All claims expressed in this article are solely those of the authors and do not necessarily represent those of their affiliated organizations, or those of the publisher, the editors and the reviewers. Any product that may be evaluated in this article, or claim that may be made by its manufacturer, is not guaranteed or endorsed by the publisher.
