# Supplementary material for: Molecular Prevalence of Anaplasma marginale and Ehrlichia in Domestic Large Ruminants and Rhipicephalus (Boophilus) microplus Ticks From Southern Luzon, Philippines
Source: Front Vet Sci. 2021 Oct 13;8:746705. doi: 10.3389/fvets.2021.746705 (PMC8548686; doi:10.3389/fvets.2021.746705)
Supplement: Supplementary file 1 [file Table_1.DOCX]

**Table S1.** Primers used for the amplification of rickettsial pathogens *Anaplasma marginale* and *Ehrlichia*

| **Organism** | **Target gene** | **Primer Name** | **Sequence**  **(5' → 3')** | **Expected size (bp)** | **References** |
| --- | --- | --- | --- | --- | --- |
| Anaplasmataceae | *groESL* | gro607F | GAAGATGCWGTWGGWTGTACKGC | 664 | Tabara et al. 2007 |
|  |  | gro1294R | AGMGCTTCWCCTTCWACRTCYTC |  |  |
|  |  | gro677F | ATTACTCAGAGTGCTTCTCARTG | 315 |  |
|  |  | gro1121R | TGCATACCRTCAGTYTTTTCAAC |  |  |
| *Anaplasma marginale* | *groEL* | AM265 | GACTACCACATGCTCCATACTGACTG |  | Ybañez et al. 2013 |
|  |  | AM1574 | GACGTCCACAACTACTGCATTCAAG |  |  |
|  |  | AMA424 | GTCTGAAGATGAGATTGCACAGGTTG | 866 |  |
|  |  | AM1289 | CCTTTGATGCCGTCCAGAGATGCA |  |  |
| *Anaplasma marginale* | *msp5* | AM-49F | GTGTTCCTGGGGTACTCCTATGTGAACAAG | 547 | Ybañez et al. 2012 |
|  |  | AM-595R | AAGCATGTGACCGCTGACAAACTTAAACAG |  |  |
|  |  | AM-211F | AAGCACATGTTGGTAATATTCGGCTTCTCA | 195 |  |
|  |  | AM-376R | AATTCTCGCATCAAAAGACTTGTGGTACTC |  |  |
| *Ehrlichia* | *dsbA* | EHL dsb-330 | GATGATGTCTGAAGATATGAAACAAAT | 409 | Sun et al. 2008 |
|  |  | EHL dsb-728 | CTGCTCGTCTATTTTACTTCTTAAAGT |  |  |

**Table S2.** PCR conditions for the amplification of target fragments of genes of rickettsial pathogens *Anaplasma* and *Ehrlichia*

| **Target gene** | **PCR condition** |
| --- | --- |
| *groESL* | $1\mathrm{st} round:\frac{{95}^{^{\circ}}C}{10 min} \to\frac{{94}^{^{\circ}}C}{30 s} - \frac{{57}^{^{\circ}}C}{30 s} - \frac{{72}^{^{\circ}}C}{1 min} 30x\to\frac{{72}^{^{\circ}}C}{5 min} \to{10}^{^{\circ}}C$ |
|  | $2nd round:\frac{{94}^{^{\circ}}C}{5 min} \to\frac{{94}^{^{\circ}}C}{30 s} - \frac{{57}^{^{\circ}}C}{30 s} - \frac{{72}^{^{\circ}}C}{30 s} 30x\to\frac{{72}^{^{\circ}}C}{5 min} \to{10}^{^{\circ}}C$ |
| *groEL* | $1st round: \frac{{95}^{^{\circ}}C}{9 min} \to\frac{{94}^{^{\circ}}C}{30 s} - \frac{{62}^{^{\circ}}C}{30 s} - \frac{{72}^{^{\circ}}C}{1.5 min} 35x\to\frac{{72}^{^{\circ}}C}{5 min} \to{10}^{^{\circ}}C$ |
|  | $2nd round: \frac{{95}^{^{\circ}}C}{5 min} \to\frac{{94}^{^{\circ}}C}{30 s} - \frac{{65}^{^{\circ}}C}{30 s} - \frac{{72}^{^{\circ}}C}{1 min} 35x\to\frac{{68}^{^{\circ}}C}{5 min} \to{10}^{^{\circ}}C$ |
| *msp5** | $1st round: \frac{{95}^{^{\circ}}C}{5 min} \to\frac{{95}^{^{\circ}}C}{30 s} - \frac{{74-68}^{^{\circ}}C}{30 s} - \frac{{72}^{^{\circ}}C}{1 min} 36x\to\frac{{72}^{^{\circ}}C}{5 min} \to{10}^{^{\circ}}C$ |
|  | $2nd round: \frac{{95}^{^{\circ}}C}{5 min} \to\frac{{95}^{^{\circ}}C}{30 s} - \frac{{74-68}^{^{\circ}}C}{30 s} - \frac{{72}^{^{\circ}}C}{30 s} 36x\to\frac{{72}^{^{\circ}}C}{5 min} \to{10}^{^{\circ}}C$ |
| *dsbA* | $\frac{{94}^{^{\circ}}C}{5 min} \to\frac{{94}^{^{\circ}}C}{30 s} - \frac{{55}^{^{\circ}}C}{30 s} - \frac{{72}^{^{\circ}}C}{30 s} 30x\to\frac{{72}^{^{\circ}}C}{5 min} \to{10}^{^{\circ}}C$ |

*Annealing with 0.2°C incremental decrease until reaching final annealing temperature at 68°C
